# Supplementary material for: Identification of photoperiod-induced specific miRNAs in the adrenal glands of Sunite sheep (Ovis aries)
Source: Front Vet Sci. 2022 Jul 22;9:888207. doi: 10.3389/fvets.2022.888207 (PMC9354845; doi:10.3389/fvets.2022.888207)
Supplement: Supplementary file 1 [file Data_Sheet_1.ZIP › Supplementary file/miRDeep2 file.zip!/miRDeep2 file/Know_Structure/SP42A3/oar-mir-143.pdf]

miRBase precursor : oar-mir-143
Total read count : 220318
oar-miR-143 read count : 218503
remaining reads : 1815

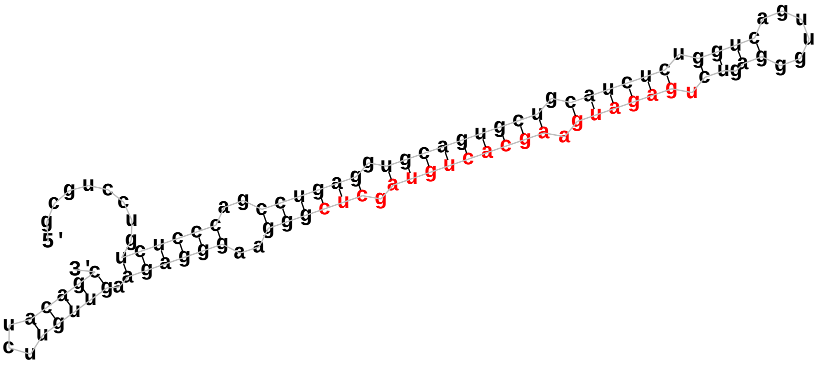

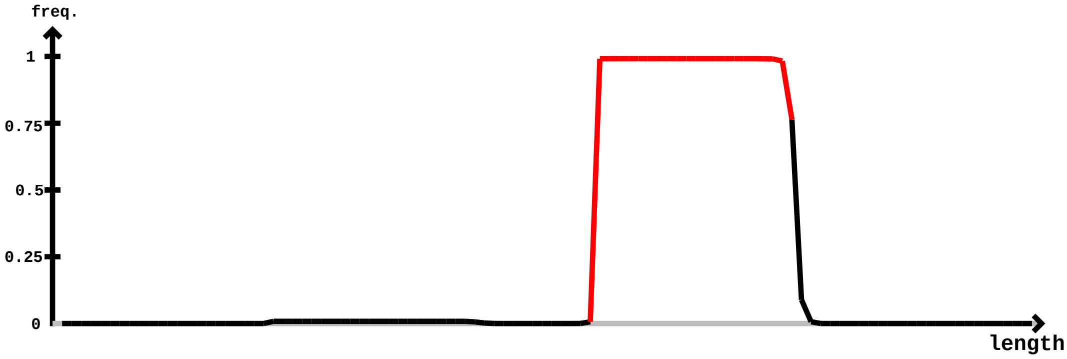

oar-miR-143

```
5'- gcguccugucucccagccugaggugcagugcugcaucucuggucaguugggagucugagaugaagcacuguagcucgggaagggagaaguuguucuacagc -3'    exp
    ........((((((..((((((.(((((((((.((((((.((((......)).)).)))))).))))))))).))))))..)))))).(((((...)))))     reads   mm   sample
    ................ccugaggugcagugcugcau.................................................................     1       0    seq
    ....................aggugcagugcugcaucucugg...........................................................     2       0    seq
    ....................aggugcagugcugcaucucuggu..........................................................     1       0    seq
    .....................ggugcagugcugcaucucu.............................................................     2       0    seq
    .....................ggugcagugcugcaucucug............................................................     8       0    seq
    .....................ggugcagugcugcaucucugg...........................................................     477     0    seq
    .....................ggugcagugcugcaucucuggu..........................................................     937     0    seq
    .....................ggugcagugcugcaucucugguc.........................................................     348     0    seq
    .....................ggugcagugcugcaucucuggucа........................................................     37      0    seq
    ....................................................gucugagaugaagcacuguagcu..........................     1       0    seq
    ....................................................gucugagaugaagcacuguagcuc.........................     1       0    seq
    .....................................................ucugagaugaagcacuguagc...........................     2       0    seq
    .....................................................ucugagaugaagcacuguagcu..........................     4       0    seq
    .....................................................ucugagaugaagcacuguagcuc.........................     46      0    seq
    ......................................................cugagaugaagcacugua.............................     1       0    seq
    ......................................................cugagaugaagcacuguagc...........................     12      0    seq
    ......................................................cugagaugaagcacuguagcu..........................     300     0    seq
    ......................................................cugagaugaagcacuguagcuc.........................     1002    0    seq
    ......................................................cugagaugaagcacuguagcucg........................     50      0    seq
    ......................................................cugagaugaagcacuguagcucgg.......................     2       0    seq
    .......................................................ugaCaugaagcacugua.............................     1       1    seq
    .......................................................ugagaugaagcacuguG.............................     2       1    seq
    .......................................................ugagaugaagUacugua.............................     1       1    seq
    .......................................................ugagaugaagcacuguU.............................     3       1    seq
    .......................................................ugagGugaagcacugua.............................     1       1    seq
    .......................................................ugagaugaagcacugua.............................     67      0    seq
    .......................................................ugagaugaagcacuguag............................     95      0    seq
    .......................................................ugagaugaagcacuguagc...........................     1762    0    seq
    .......................................................ugagaugaagcacuguagcu..........................     48031   0    seq
    .......................................................ugagaugaagcacuguagcuc.........................     147540  0    seq
    .......................................................ugagaugaagcacuguagcucg........................     18074   0    seq
    .......................................................ugagaugaagcacuguagcucgg.......................     1417    0    seq
    .......................................................ugagaugaagcacuguagcucggg......................     12      0    seq
    ........................................................gagaugaagcacuguagcu..........................     12      0    seq
    ........................................................gagaugaagcacuguagcuc.........................     29      0    seq
```

oar-miR-143

gcguccugucucccagccugaggugcagugcugcaucucuggucaguugggagucugagaugaagcacuguagcucgggaagggagaaguuguucuacagc			
....gagaugaagcacuguagcucg........................	12	0	seq
....gagaugaagcacuguagcucgg.......................	1	0	seq
....agaugaagcacuguagcu..........................	1	0	seq
....agaugaagcacuguagcuc.........................	3	0	seq
....agaugaagcacuguagcucgg.......................	10	0	seq
....agaugaagcacuguagcucggg......................	1	0	seq
....gaugaagcacuguagcu..........................	2	0	seq
....gaugaagcacuguagcucgg.......................	3	0	seq
....gaugaagcacuguagcucgggaa....................	4	0	seq
